# The Dislocation- and Cracking-Mediated Deformation of Single Asperity GaAs during Plowing Using Molecular Dynamics Simulation

**DOI:** 10.3390/mi13040502

**Published:** 2022-03-24

**Authors:** Baozhen Li, Jianyong Li, Wengang Fan, Tong Xuan, Jinhuan Xu

**Affiliations:** 1 School of Mechanical, Electronic and Control Engineering, Beijing Jiaotong University, Beijing 100044, China; 17116367@bjtu.edu.cn (B.L.); jyli@bjtu.edu.cn (J.L.); 16116345@bjtu.edu.cn (T.X.); 16116346@bjtu.edu.cn (J.X.); 2 Key Laboratory of Vehicle Advanced Manufacturing, Measuring and Control Technology, Ministry of Education, Beijing 100044, China

**Keywords:** molecular dynamics simulations, single asperity GaAs, plowing, deformation behaviors, crack propagation

## Abstract

This work simulates the plowing process of a single asperity GaAs by diamond indenter using molecular dynamics simulations. The deformation mechanism of asperity GaAs is revealed by examining the topography evolution and stress state during the plowing. This work also investigates the origin of the influence of asperity size, indenter radius and plow depth on the deformation of the asperity GaAs. We observed the initiation and propagation of cracks up to the onset of fracture and the plastic activity near the indenter, obtaining more information usually not available from planar GaAs in normal velocity plowing compared to just plastic activity. The simulations demonstrated the direct evidence of cracking in GaAs induced by plowing at an atomic level and probed the origin and extension of cracking in asperity GaAs. This finding suggests that cracking appears to be a new deformation pattern of GaAs in plowing, together with dislocation-dominated plasticity modes dominating the plowing deformation process. This work offers new insights into understanding the deformation mechanism of an asperity GaAs. It aims to find scientific clues for understanding plastic removal performed in the presence of cracking.

## 1. Introduction

Gallium arsenide (GaAs) has gained significant attention as a semiconductor material due to its wider energy bandgap, high frequency, higher electron mobility and other characteristic advantages compared to Si [1,2]. Therefore, it is widely used in communication devices and integrated microcircuits. In addition, it can also be used as a structural material for a variety of integrated micromechanical microsensors and microactuators with more technical advantages [3]. Therefore, nano and sub-nano manufacturing is gaining increasingly more attention [4,5]. However, with the remarkable development of microelectromechanical systems (MEMS), the problem of frictional failure has become a vital issue limiting the performance of MEMS devices, while sliding contact is a typical situation causing MEMS device failure [6]. The failure contact occurs between the rough surfaces of the asperities of nanoscopic dimensions. The wear phenomenon usually involves the contacts between two asperities on the surface, resulting in breaking old and forming new atomic bonds and plastic deformation in the contact zone. The centrality of a single asperity in the basic micromechanical reaction of contact between two rough surfaces has long been appreciated [7]. Investigating the processes that occur at the atomic level during the sliding contact of rough asperities is essential for fundamental tribological issues, including contact formation, friction, plastic deformation, wear and fracture. This paper provides a theoretical basis for the structural design of MEMS devices by focusing on the deformation behavior of single asperity.

Many scholars have worked in different directions in the study of atomic and close-to-atomic-scale manufacturing, which also includes extensive experiments and simulations of AFM indentations and scratches [8,9,10,11,12]. In addition, some scholars have conducted a more detailed MD study on the deformation behavior of GaAs. However, all existing studies focus on the case of smooth surfaces, conducting MD simulations of the cutting process of GaAs on smooth surfaces to explore a series of phenomena in the deformation process [13,14,15]. In fact, from a microscopic point of view, the indenter is in contact with the coarse surface, both in the actual contact case of power devices and in the scratching experiments [16]. In contrast, the importance of asperity GaAs in the scratching behavior for the underlying micromechanical response has long been neglected. For example, in MD simulations of crystal deformation, the deformation behavior of a roughened workpiece is significantly different from that of a flat workpiece [17]. Therefore, existing studies remain poorly understood on the effect of the mechanical loads’ frictional behavior in plowing on the microscopic deformation of the single asperity GaAs. There is still not enough evidence to firmly explain the range of physical phenomena in the deformation process. Moreover, the microscopic deformation mechanisms are not yet fully understood [14,18].

In GaAs nanoscratching experiments, evidence of dispersion dislocations, plastic deformation and cracks in the scratch zone is frequently reported [18]. Although cracks in brittle materials during scratching are expected and confirmed in scratching experiments, it is not common in simulations [19]. It should be noted that the occurrence and propagation of cracks have never been observed in simulations of the regular velocity plowing of GaAs. The only reported cracks in MD simulations of GaAs scratching were under high-speed tool impact, which did not correspond to actual working conditions at lower speeds [20]. Furthermore, the occurrence of cracks has never been reported for rough GaAs. At the same time, the microscopic mechanisms of crack emergence and propagation in GaAs are not entirely understood and the contribution of the crack to deformation behavior is not yet clear. Additionally, studies of GaAs in plowing simulations have shown that the deformation process is always accompanied by the formation of dislocations, stacking faults and phase transformation [21,22]. The mechanical deformation response of GaAs to contact damage has attracted extensive attention [23]. However, the crack initiation and propagation event we observe in this study is an extraordinary rare physical phenomenon in nanoscratch simulations of GaAs, and we believe the geometrical properties of the sample may have influenced it. Therefore, the crack onset and propagation mechanism needs to be further clarified.

MD simulations are expected to play an essential role in elucidating these mechanisms. They overcome the limitations of the experimental scale. They enable the study of material deformation behavior occurring within a few picoseconds and at the nanoscale, making it possible to investigate the microscopic nature of material frictional deformation based on computational simulations. They allow for a better decoupling of the factors influencing the deformation behavior under realistic conditions; thus, allowing for a more intrinsic mechanical response and an analysis of the material frictional deformation. They play an essential role serving as a reliable tool for analyzing the internal mechanisms of experimental phenomena at the atomic scale. MD plow simulations can be used as a model experiment to understand the GaAs’ deformation behavior and crack formation mechanism during the plowing process.

## 2. Materials and Methods

**Figure 1:**
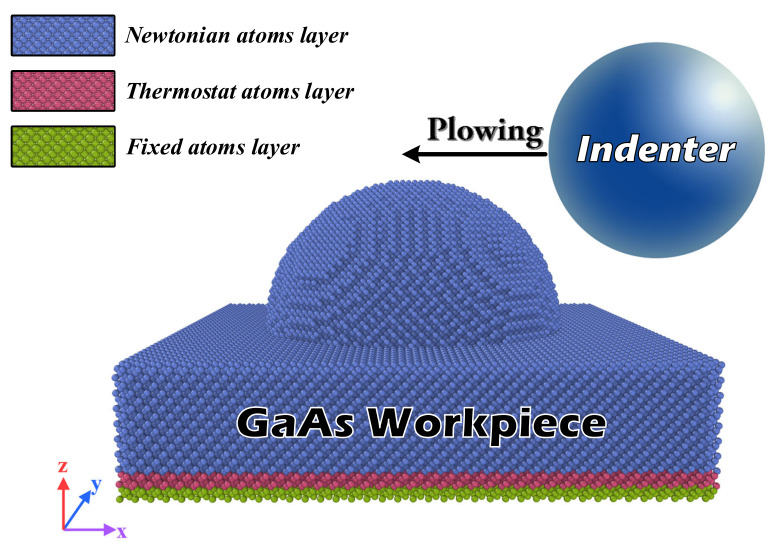
Schematic of the plowing model with a spherical asperity on GaAs substrate.

Figure 1 demonstrates the MD simulation model of the plowing process. For a detailed parametric study, the idealized setup of a rough hemispherical peak on the substrate plane was used as the single asperity. The hemispherical surface roughness peak had the same initial topography as the spherical indenter in a typical asperity–asperity shear MD simulation. The x, y and z directions of the model were (100), (010) and (001), respectively. The size of GaAs substrate was 22 nm × 22 nm × 5 nm and the rough hemispherical peak was located in the center of the substrate surface with radii ranging from 2 nm to 7 nm. The whole system contained 109,512 substrate atoms. The substrate was divided into three parts, distinguished according to the color in Figure 1. The fixed layer atoms, the thermostatic layer atoms and the newton layer atoms were shown in green, pink and blue, respectively. The fixed layer atoms at the bottom of the substrate were fixed to prevent the substrate from moving. The atoms in the thermostatic and Newton layers follow Newton’s second law of motion. The atoms next to the boundary region were the thermostatic layer atoms and the microregular ensemble (NVE) was used. The velocity Verlet algorithm was used to solve the equation of motion and the initial temperature was set to 300 K. The temperature was controlled by a Nose–Hoover heat bath. The free boundary conditions were used in the z-direction and the periodic boundary conditions were used in the x and y directions. In these MD simulations, the plowing speed was 100 m/s as in previous studies [24]. Although this high plowing speed was unrealistic, this setting helped to keep the computational time at a reasonable value for computational efficiency. The detailed parameters of the simulation setup are shown in Table 1.

In order to produce a more uniform resultant stress field and provide an extended elastoplastic regime before cracking was initiated, it was beneficial for us to use a spherical indenter [25]. Due to the significant difference in macroscopic hardness between diamond and GaAs, the indenter was considered as a rigid body without deformation. In this work, the force field consisted of a hybrid scheme, in which the interactions between GaAs atoms and the cross interaction between the indenter and GaAs atoms were described by the Tersoff potential function [26] and the Ziegler–Biersack–Littmark (ZBL) potential function [27], respectively. This study was implemented using the open-source atomic/molecular massively parallel simulator code (LAMMPS) [28]. The instantaneous atomic image during the plowing process was obtained by Open Visualization Tool (OVITO) [29], which is an open MD visualization tool. After the system relaxed, the indenter was pushed to plowing along the x-direction.

## 3. Results and Discussion

### 3.1. Analysis of the Plowing Process of Single Asperity GaAs

**Figure 2:**
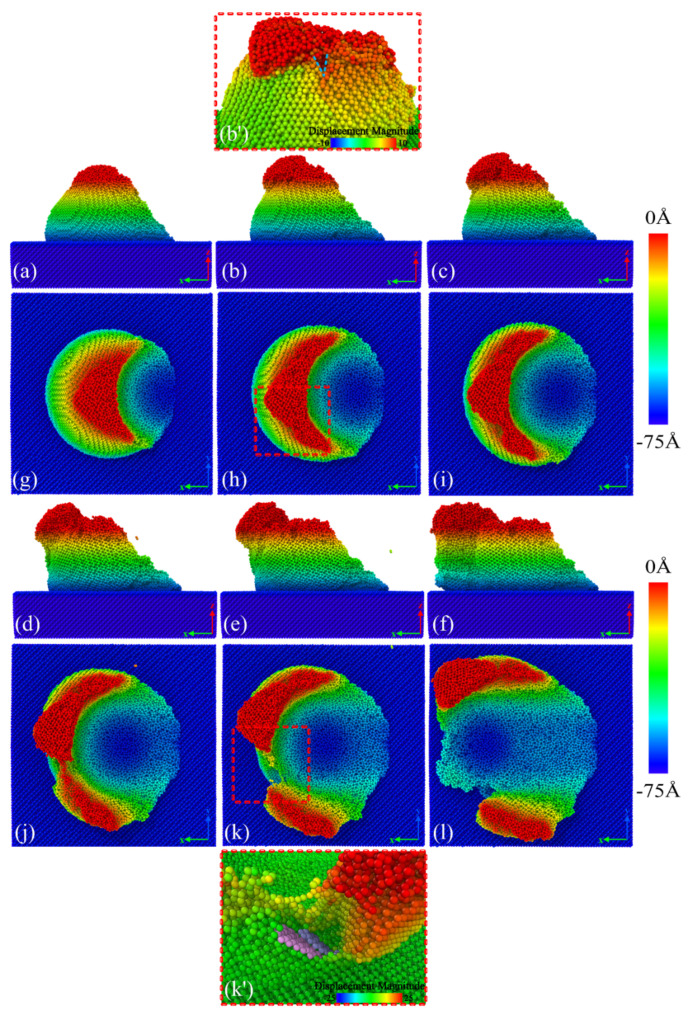
Deformation behaviors of single asperity GaAs(001) during plowing at (**a**,**g**) 7 nm, (**b**,**h**) 9 nm, (**c**,**i**) 10 nm, (**d**,**j**) 12 nm, (**e**,**k**) 13 nm and (**f**,**l**) 18 nm plowing distance, respectively. (**a**–**f**) is the main view and (**g**–**l**) is the top view. Each atom was colored according to its height of normal direction. (**b’**) and (**k’**) show local views of the dashed part, whose atoms were colored according to the displacement magnitude. In (**b’**), the blue V line indicates the place where the crack started. In (**k’**), the pink and blue atoms highlight the smooth surface where the crack formed.

From the variation of the atomic lattice state, the deformation mechanism of the GaAs peak was studied utilizing the atomic configuration. Figure 2 shows the asperity deformation of the whole process at different plowing distances. The height of asperity was 7 nm and the indenter radius was 6 nm. Here, we defined the plow depth as the distance normal to the indentation from the asperity top. We set the plow depth to 7 nm to highlight the features of the asperity in the deformation without affecting the substrate. The contact, deformation and material removal occurred in aggregation, plowing and separation between single asperity surfaces. It can be seen that the lattice near the contact area had varying degrees of deformation, indicating that the energy was stored in the lattice in the form of lattice strain energy. With the indenter movements, the deformation first occurred at the rough peak atoms in contact with the indenter, as shown in Figure 2a. The deformation of the atomic lattice in the contact squeeze region led to an increase in potential energy. When the strain energy stored in the deformed lattice exceeded the threshold value, the atoms would rearranged in the form of low-lattice energy to release the lattice energy. As shown in Figure 2b, the plastic deformation intensified with the further contact between the indenter and the asperity. At the same time, the image shown in Figure 2b’ illustrates the signs of the appearance of a crack. The initial crack formed at the end of the amorphous region and extended into the single crystal. We examined the complete process of surface roughness peaks undergoing deformation, wear and tear until they were destroyed. In particular, the lattice structure was seriously damaged, and the amorphous structure was formed by disorderly mixing at the top of the rugged peak. The atoms at the peak were less stable than the atoms in the equilibrium inside the workpiece matrix, could withstand less load and were more prone to changes in atomic position. The workpiece material’s structure caused this. In addition, as shown in Figure 2g–l, the rough peak was torn under the action of the indenter extrusion, the crack gradually expanded and moved to both sides, the rough peak moved to both sides and the front end under the action of indenter plowing was extruded to both sides of the rough peak and, eventually, formed a thin sheet-like structure of wear debris. GaAs atoms that detached from the ideal lattice position during plowing were identified as debris atoms. Some scholars have adopted a similar method to identify wear debris. The morphology showed a tearing of the material from a weak point, which accelerated the movement of the stripped rough peak atoms towards the smooth surface of the workpiece. The pile-up of rough peaks due to plastic flow was also evident at the front of the indenter. Dissimilar to the plowing of smooth GaAs, where the deformation occurred in the substrate, however, the deformation of asperity GaAs occurred mainly within the rough peaks. Plow simulations indicated that crack emergence was difficult in smooth surface crystal structures. However, deformation accompanied by cracking events on the surface was plausible, as verified in many experimental studies of plowing [30,31]. For the first time, we found that not only did plastic flow occur during the extrusion deformation of rough peaks, but cracks also developed as the starting point of fractures. The cracks propagated from the side of the plow path in a curved shape, ending roughly perpendicular to the plow path, forming a smooth surface on either side of the crack, as shown in Figure 2k’. This created a deformation mechanism that was significantly different from that of planar GaAs. It should be noted that the material removal pattern was influenced by cracks, which did not penetrate through the substrate material but were instead obstructed at the interface between the rough body and the substrate, as shown in Figure 2f,l, which resulted in the appearance of cracks that did not damage the substrate.

**Figure 3:**
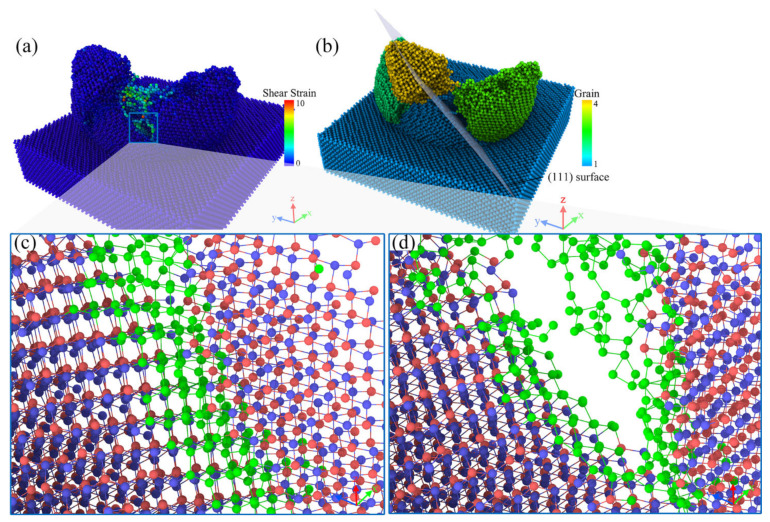
Screenshots of the extension of the cracks. (**a**) shows the distribution of atomic shear strain at 12 nm plowing distance, at which point (**b**) shows the workpiece in a polycrystalline state, with the translucent (111) fracture surface being the dissociated surface of the GaAs. (**c**,**d**) show the evolution of the atomic structure of the crack formation process at 11 nm and 12 nm plowing distances, respectively.

In order to investigate the crack formation process, we concentrated on the crack extension during the plowing process at the 11 nm to 12 nm moment, as shown in Figure 3. In Figure 3a, it can be seen that all the atoms of the workpiece subjected to shear strain were distributed around the crack. We focused on the behavior of the atoms subjected to shear strain during crack formation, as shown in Figure 3c,d, where the atoms in the green part of the diagram were the atoms subjected to shear strain in the diagram. The remaining Ga and As atoms were colored red and blue, respectively. The bonds between the atoms were shown in the diagram. An empirical cutoff distance of 0.3 nm was used to determine whether a bond was broken.

**Figure 4:**
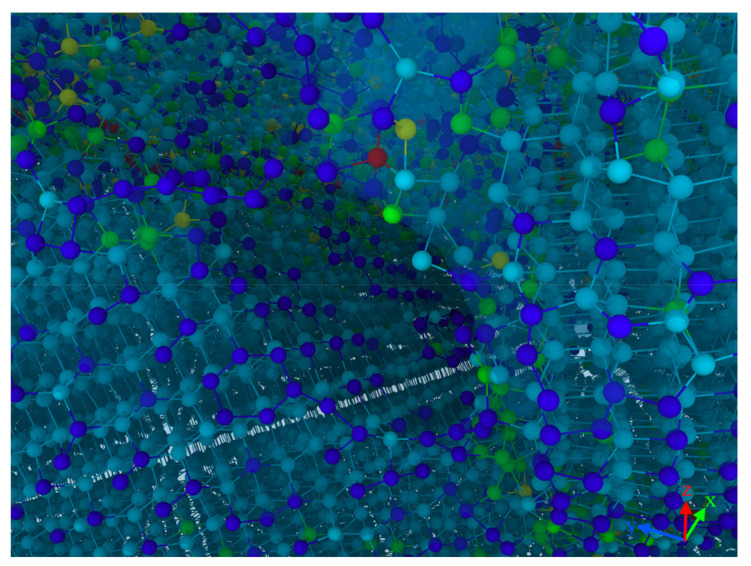
A snapshot of the atomic structure near the crack tip, with the atoms colored according to the coordination numbers.

In Figure 3c,d of the crack formation section, we could clearly observe that the atoms subjected to shear strain as shown in the figure were subjected to tensile displacement, leading to a local covalent bond breakage, with the crack extending downwards. The right side of the crack was pulled away from the workpiece and, eventually, the material was pulled apart. It can be assumed that the local tensile stress was greater than the threshold value for crack formation. At the same time, the lattice lines on both sides of the crack remained straight without bending, as can be seen more clearly in Figure 4.

As the crack propagated, the material was split into polycrystals by thin amorphous bands and cracks, as shown in Figure 3b. Different grains were shown using different colors and we could observe grains with different crystallographic orientations, which were shown along different crystal orientations. The crack was flanked by single crystals with a neat structure. Based on the crack extension direction, the fracture plane was the (111) crystal plane shown in Figure 3b. This was due to GaAs in the (111) crystallographic plane having the largest atomic surface density and the furthest crystallographic spacing, as shown in Table 2. At the local level, these were the preferred planes for crack propagation. This resulted in the (111) crystal plane having minimal interactions and stronger atoms on the crystal plane. At this point, cracks were more likely to form along the (111) plane, and produced a smooth fracture surface. This phenomenon was consistent with the macroscopic scale, where the (111) crystal orientation was the cleavage plane of GaAs [32]. The above confirmed that cracks were not due to plastic flow.

In order to elucidate the atomic states in the vicinity of the crack, a local view of the crack region was observed as an example of this crack formed at the 12 nm plow moment in Figure 3. Here, we focused on the distribution of coordination numbers on both sides of the crack, as this related to the state of the lattice, as shown in Figure 4. The atoms were colored according to their coordination number (CN), which was used to characterize the phase transformation of GaAs. When the coordination number was less than or equal to three (CN ≤ 3), there were dangling bonds on the GaAs surface, which were used to represent the surface atoms (dark blue atoms). The intrinsic cubic zinc-blende structure atoms with CN = 4 were primitive with a perfect lattice (light blue atoms). The atoms with CN = 5 (green atoms), CN = 6 (yellow atoms) and CN = 7 (red atoms) were the intermediate structure, the rock-salt structure and the amorphous structure, respectively, in which the phase transformation took place. A light blue translucent surface was constructed in the visualization software to facilitate the distinction between the hemispherical surface and the newly formed cracked surface. As seen in the diagram, in addition to the surface atoms with CN = 3, the atoms with CN = 3 were also regularly arranged on the fracture surface where the crack was formed. It is to be noted that the dark blue atoms had dangling bonds. These atoms with dangling bonds on the crack face originated from the breakage of the Ga–As bonds formed along the adjacent (1 1 1) planes, so that a smooth fracture surface was observed (see Figure 4 or inset of Figure 3d or Figure 2k’). The atoms on the crack face did not undergo phase changes, dislocations or homogeneous plastic behavior, and the stress could not be released. As a result, the crack extended more quickly and could be seen as a neat single crystal on the crack face, as could also be seen in the grain-splitting diagram. We believe that the reduction in stored elastic strain energy was more significant than the energy required to form two new surfaces due to cracking. The edge crack extended in a brittle manner and, therefore, the cracked surface was very flat and smooth.

**Figure 5:**
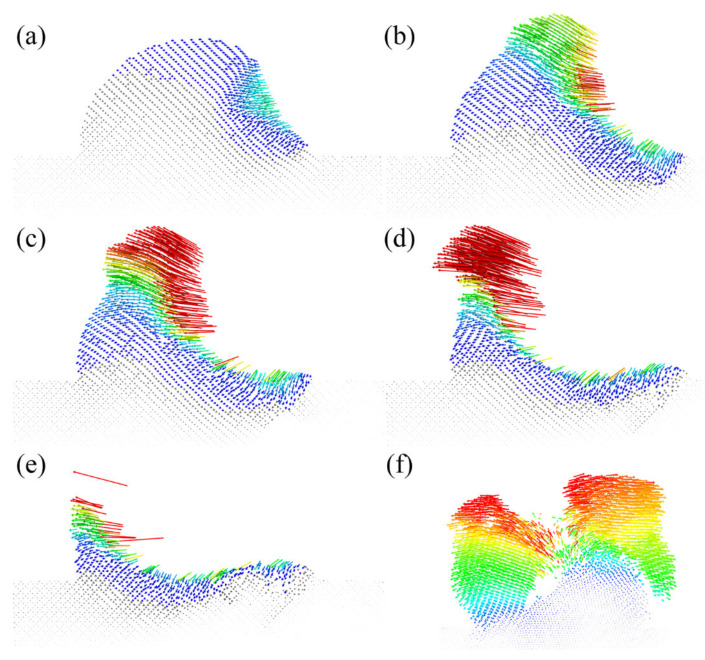
Atomic displacement vector diagram in different plowing stages; (**a**–**e**) represent the plowing distance of d = 4 nm, 7 nm, 9 nm, 12 nm and 14 nm. Additionally, (**f**) shows a view of the crack plane at 12 nm distance, demonstrating the trajectory of the atoms on either side of the crack. The direction of the arrow indicates the moving direction of atoms and the color of the arrow indicates the magnitude of the displacement.

**Figure 6:**
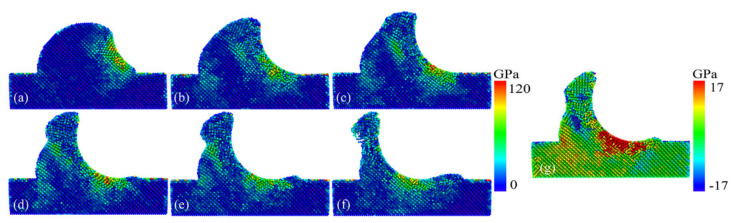
Distribution of von Mises stresses at plowing distance of (**a**) 4 nm, (**b**) 6 nm, (**c**) 7 nm, (**d**) 9 nm, (**e**) 10 nm and (**f**) 12 nm and (**g**) the hydrostatic stress at the highest tensile stress value.

As shown in Figure 5, we could see the atomic displacement trajectories during different plowing stages. Thus, with the help of atomic displacement levels, it was possible to carve out the trajectory of the chip formation. First, as shown in Figure 5a, a small amount of the lattice structure was disrupted at the edge of the interface between the indenter and the rough peak, at which point the atoms in the contact area moved along 45° towards the rough peak. As the rough peak was further squeezed, more GaAs atoms were involved in the movement. However, this phenomenon could only last for a while until the plowing distance reached 9 nm. From Figure 5c, we found that the atomic trajectory could be divided into two categories, and there was an apparent boundary between the two trajectories. After that, some atoms moved continuously toward the interior of the substrate, while more atoms began to move to the top of the rough peak. This was also verified in Figure 5d, where more atoms of the rough peak moved upwards to form chips. Thus, the formation of chips at the front of the indenter resulted from the plastic flow caused by extrusion. Moreover, a small number of atoms moved downward and backwards due to extrusion, resulting in subsurface damage of the substrate. As the plowing proceeded, many chips were produced at the front of the rough peak due to the breaking of the atomic bonds. In addition, it was found that some elastic recovery occurred on the scratched surface after the indenter moved, which was due to the movement law of the workpiece atoms at the lower end of the indenter moving backwards in an arc. This feature allowed the scratched surface to have shallow subsurface damage compared to plowing on smooth surfaces. Meanwhile, after the plowing was completed, the indenter was removed through some rough peaks to form a flatter surface. At the same time, we observed the atomic trajectory in the plane where the crack was located at the moment of fracture, as shown in Figure 5f, and we found that the atoms on both sides of the crack did not move symmetrically and did not move the same distance. Many atoms on the left side of the crack moved behind the initial position, while the atoms on the right side of the crack moved horizontally towards the initial position. This correlated with the stress level experienced at this moment and was also the leading cause of flow damage, as shown in the green area on the left side of Figure 6e.

In order to observe the complex stress state of the cracking behavior of the rough peak surface, we described the state of the von Mises equivalent stress during the deformation of the model and the hydrostatic stress distribution at the maximum tensile stress, respectively. The von Mises stress distribution is suitable for evaluating the inelastic deformation behavior of local atoms and can be used to analyze the most dangerous areas [33]. From the analysis of the stresses within the spherical asperity, we knew that the high-stress region was first concentrated in part in contact with the indenter, as shown in Figure 6a. When the stress applied by the indenter exceeded the yield strength of the workpiece material, the material underwent plasticizing behavior. As the asperity was deformed, from Figure 6a–c, we observed that the atoms inside the asperity were subjected to an increasing degree and a range of stresses, continuously accumulating deformation energy. The stress distribution was divided into two central regions: the substrate region under the indenter and the rough peak region under the chips. As the indenter was pushed, a higher stress region appeared inside the rough peak, which gradually spread towards the opposite side of the surface, as shown in Figure 6b–e. Until the rough peak crack propagation event entirely occurred, all of the strain energy accumulated in the asperity immediately below the chips was released, as shown in Figure 6e,f. The atomic stress in the green region below the chips decreased to 0. 

In the hydrostatic stress distribution, we observed that the asperity that was in compressive stresses appeared on the side in contact with the indenter, while the front side of the asperity was in tension, as shown in Figure 6g in the region of tensile stress (blue atoms). We believe that tensile stresses promoted surface cracking, leading to the emergence of surface tensile cracks, which was consistent with the distribution of hydrostatic stresses. Indeed, the area of tensile stress distribution coincided with the location of crack emergence. At the same time, the local tensile stress field guided the crack growth along a defined path. The state of stress distribution was significantly different from that in planar GaAs scratching, where tensile stresses were distributed deep inside the workpiece. However, the tensile stress was at too low a level, with a maximum value of only 3.40 Gpa [20] in a similar scale model, where cracks were difficult to form. In this paper, we believe that, thanks to the reasonable shape of the asperity which led to a strong material flow close to the indenter side, the extrusion and friction in the subsurface also brought about severe stresses. The maximum value of tensile stress was 18.14 Gpa, and the cracks were all located near the tensile stress zone, so it can be assumed that the local tensile stress was greater than the threshold of crack formation.

**Figure 7:**
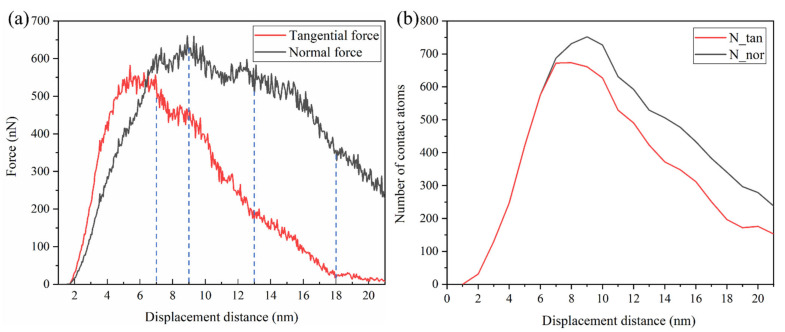
(**a**) The force–plowing length curves and (**b**) the number of contact atoms in the normal and tangential directions.

The tangential and normal force between the indenter and the GaAs under different displacement distances are shown in Figure 7a. When the plowing distance was 5 nm, the friction first reached a stable state, where the shear stress dominated and fluctuated slightly around 550 nN during the next 2 nm of plowing. A certain amount of the stored elastic energy at the asperity junction accompanied the plowing. Until the plowing distance reached 7 nm, the tangential force decreased sharply away from the steady state, while the normal force continued to increase and also reached its first peak value. This was different from the case of scratching a flat workpiece, which was caused by the variation in the actual number of atoms in contact between the indenter and the asperity in the normal and transverse contributions, as shown in Figure 7b. At this point, the nucleation of dislocations was found here, where the load was greater than the yield point and permanent plastic deformation occurred. 

After a short drop, the tangential force was followed by a very short and steady fluctuation. Then, at a plowing distance of 9 nm, the tangential force was followed by a dramatic drop event. We knew that the pop-in may be associated with a critical deformation level. Moreover, this was when cracks were observed at the contact interface, as shown in Figure 2b’. Our computational simulations showed that the sudden drop in tangential force at a plowing distance of 9 nm was caused neither by a high-pressure phase transition nor by dislocation activity, reflecting the appearance of a crack in the asperity surface instead. There was possibly one mechanism that could account for this phenomenon: the activation mechanism of the crack causing the pop-in event. The cracking that occurred when the fracture strain threshold was exceeded released the deformation energy, resulting in wave troughs of the tangential force. The abrupt drop in force on the curve marked the point at which the crack growth was initiated. Scratching experimental studies by Wang et al. [34] in metallic glass have also shown that sudden pop-ins in friction curves were associated with crack formation. As a result of crack formation, the indenter lost partial contact with the single asperity and the normal force dropped in fluctuation.

Until the plowing distance reached 13 nm, the crack was fully formed in the vertical direction, as shown in Figure 2k’. Then, with the continued pushing of the indenter in the forward direction, the cracks continuously expanded along the lateral direction. As the contribution to the tangential force gradually decreased, the tangential force started to decrease slowly and uniformly. After a plowing distance of 18 nm, the decline in normal and tangential forces slowed down further as the indenter gradually moved away from the single asperity.

**Figure 8:**
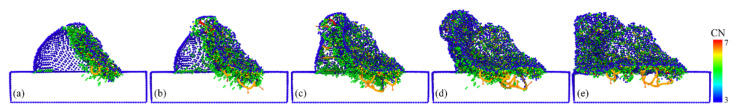
Plastic deformation during plowing process; (**a**–**e**) represents the plowing distance of d = 7 nm, 8 nm, 10 nm, 14 nm and 21 nm.

To further study the deformation and removal behavior, we analyzed the microstructure evolution of GaAs during the plowing process, as shown in Figure 8. Figure 8 shows the detailed process of the phase transformation and dislocations coexisting in GaAs during processing. Figure 8a implies the initial position of dislocation nucleation, with the preferred sites for dislocation nucleation occurring at the point of maximum pressure, as shown in Figure 6c. As the indenter progressed in the plowing direction, an external force was applied to the perfect zinc-blende structure, which resulted in a displacement of the GaAs atoms. The distribution of coordination numbers during the plowing showed dislocation nucleation and a large number of intermediate atoms and very few atoms of the amorphous and rock-salt structures appeared on the asperity closest to the indenter. In marked contrast to a flat surface plow, the dislocation distribution existed not only in the substrate below the indenter, but it also remained inside the rough peaks in front of the moving indenter. In contrast to plowing on a flat surface, the nucleation and propagation of dislocations occurred mainly within the asperity and in the substrate in contact with the indenter. In planar plowing, the deformation energy for crack eruption was released by the nucleation of dislocations, as seen from the pop-in event of the plowing force in Figure 7. Therefore, this may be why the crack emergence was not easily detected in planar plowing-related simulation studies.

### 3.2. The Effect of the Asperity Size

In order to quantitatively understand the effect of asperity size on deformation behavior, we established a series of hemispherical asperities with different geometrical dimensions. Under the same indenter size condition, three samples with a single asperity were plowed. The asperity was selected to be small and extremely small compared to the size of the indenter. The asperity radius was 2 nm, 4 nm and 7 nm, respectively. The plowing depth was consistent with the height of asperity to ensure the rationality of deformation behavior.

**Figure 9:**
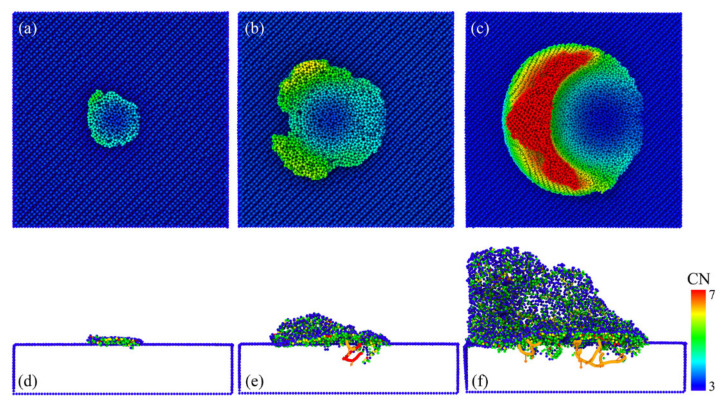
(**a**–**c**) and (**d**–**f**) represent the surface morphology at significant cracks and the defect structure at plow completion at asperity radii of 2 nm, 4 nm and 7 nm, respectively.

Figure 9 shows a top view of the surface morphology of different asperity sizes at a significant crack and a cross-sectional view of the defect structure after the plow. In the diagram, we removed the zinc-blende structure atoms with a coordination number of four. In addition, the different types of dislocations present within GaAs were extracted and identified with the aid of an automatic dislocation extraction algorithm (DXA) [35].

It can be seen that there were apparent differences in the deformation behavior of rough peaks with different sizes after plowing. From the height of the “residual roughness peak” left on the substrate surface after plowing, it can be seen that the smaller the particle size of the roughness peak, the easier it was to obtain the surface with lower surface roughness under the same size of indenter. In addition, with the increase in the rough peak size, the deformation of GaAs became more apparent, and the number of phase transition atoms and dislocation lines increased. This was because the contact area between the indenter and the rough peak increased with the rough peak radius.

It was observed from Figure 9a,d that the rough peak with a radius of 2 nm was flattened after plowing, the substrate had no phase transformation and no evidence of dislocation movement was found, which was caused by the contacting workpiece that was only a microsphere and the tiny contact scale [36]. Therefore, the size of the rough body also determined the type of internal defect [37].

Cracks such as the ones observed in GaAs were not observed in the case of ultra-small rough peaks (r = 2 nm). However, the rough peak with a height of 4 nm showed a significant crack in front of the indenter, and the rough peak with a height of 7 nm showed two cracks on either side of the advancing direction of the indenter. It was also observed that for the ultra-small rough peak (r = 2 nm), the lattice of the substrate connected with the rough body could maintain a perfect lattice after plowing. Because the deformation happened due to lattice transformation localized without disturbing the substrate material. The deformation was entirely determined by the plastic flow caused by the phase transformation.

**Figure 10:**
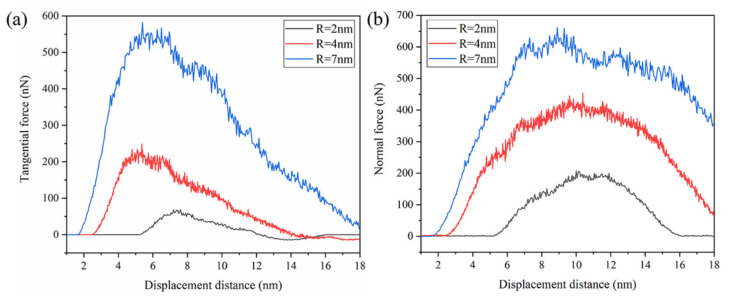
Force–displacement curve of plowing with different asperity sizes in the (**a**) tangential direction and in the (**b**) normal direction.

The force increased first and decreased with an increasing plowing distance, showing a similar evolution of irregularities under various rough peak scales. As shown in Figure 10a, at the plowing distance of 6 nm, the tangential force curve of the rough peak with a radius of 4 nm fluctuated violently with the nucleation of a dislocation loop with the Burgers vector = 1/2 [1,2,3,4,5,6,7,8,9,10]. After plowing at a distance of 0.5 nm, the force curve decreased significantly due to the formation of a crack and a large number of protruding chips piled-up above the asperity. Due to the reduced scale of the rough body, the dislocation nucleation and cracking that occurred were very close together and, therefore, did not display a greater decrease in the tangential force curve. When the plowing distance reached 10.5 nm, the friction curve decreased due to a significant crack event. Because of the small size of the asperity, the distribution of microcracks was dense. Finally, only one crack was observed and the crack extended along the forward direction of plowing, which could not effectively hinder the forward movement of the indenter. Therefore, the tangential force curve decreased gently and had a slight fluctuation. The tangential force curve shows a significant difference for a rough body with a radius of 2 nm. Although the stress reached the yield limit, it was limited to the size of the rough peak. There was no sign of dislocation nucleation and fracture event, and there was no significant pop-in event in the stress plot.

**Figure 11:**
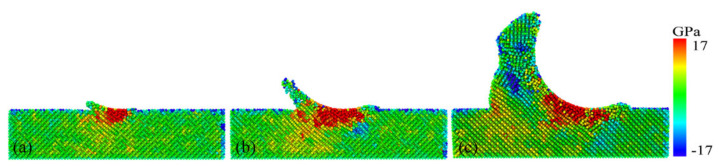
Hydrostatic pressure distribution at different asperity sizes of (**a**) 2 nm, (**b**) 4 nm and (**c**) 7 nm.

Further insight into the hydrostatic pressure distribution of the workpiece was obtained. For the case of Figure 11a, the smaller size of the asperity caused only a few atoms to come into contact with the indenter during plowing. GaAs underwent a phase transformation from a zincblende structure to a six-coordinated rock-salt structure at a compressive stress of about 17 GPa [38]. At the atomic scale, if only a few atoms of an asperity are in contact, local stresses in these regions may be abnormally high, leading to a high degree of local plastic deformation and heat generation, and even possibly local melting among asperities [39]. Hence, the minor asperity case with a radius of 2 nm was subjected to a higher hydrostatic pressure. At the same time, the force on the small rough peak was relatively concentrated, which was more prone to deformation. Note that deformation caused by both dislocations and cracks released local strain energy and stresses caused by lattice bending and elastic deformation. Therefore, the stress state could be severe in small roughness workpieces, where there were few or no dislocations and cracks.

### 3.3. The Effect of the Indenter Size

**Figure 12:**
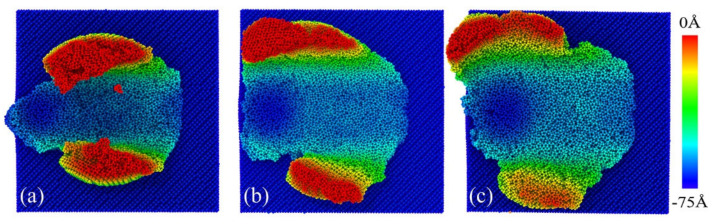
An indenter of various sizes of plows; the top view image of the rough GaAs. The indenter size was (**a**) 9 nm, (**b**) 12 nm and (**c**) 14 nm, respectively. The height of the rough peak of GaAs was 7 nm and the plowing depth was 7 nm. Atoms were colored by heights in z-direction.

In order to investigate the effect of the indenter radius on asperity deformation, we selected three indenters of different sizes to conduct the simulations. We were convinced that changing the indenter size would affect the deformation behavior. Figure 12a–c shows the instantaneous atomic configuration after plowing with indenter sizes of 9 nm, 12 nm and 14 nm, respectively. At an indenter radius of 4.5 nm, the formed chip at the bottom of the advancing direction of the indenter had a more debris-like appearance, as shown in Figure 12a. It was well understood that there were more rough peak atoms on the moving track of the indenter after plowing, which was regarded as surface roughening. More rough peak atoms remained in their original position on the single plowing track borne by a single indenter, which meant that the surface had not improved. We knew that the smaller the indenter size, the lower the stress range and the higher the stress concentration. As the indenter size increased, the spacing of the cracks widened, while the fracture dominated by the primary cracks resulted in no chip formation ahead of the indenter tip.

**Figure 13:**
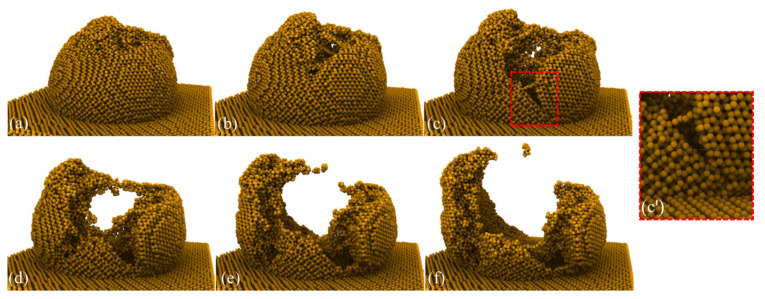
The rough GaAs morphology images show surface deformation accompanied by the formation and propagation of V-shaped cracks at the plowing distances of (**a**) 10 nm, (**b**) 11 nm, (**c**,**c’**) 12 nm, (**d**) 13 nm, (**e**) 14 nm and (**f**) 15 nm in the case of the indenter diameter of 9 nm.

**Figure 14:**
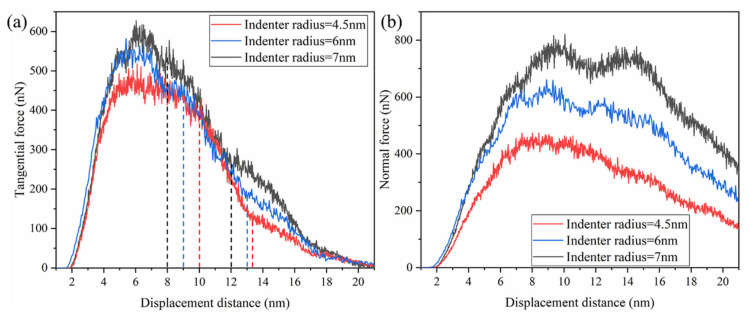
Variation of (**a**) the tangential forces and (**b**) the normal forces in various indenter radii against plowing distances.

More significant cracks appeared during plowing with an indenter radius of 4.5 nm, as shown in Figure 12a and Figure 13. With the reduction in the indenter size, higher stresses could be provided to initiate cracking. At a 10 nm plow distance, microcracks appeared on the surface of the rough peak, as shown in Figure 13a. This crack was formed at the intersection of the (111) slip band, which we explored in detail in Section 3.1. A median crack was visible where two (111) planes intersected in the asperity, as illustrated in Figure 13b. As a result, the tangential force started to decrease at a specific rate. The slope of this stage was constant, as can be seen in the graph as shown in Figure 14a. Additionally, note that this surface cracking became less pronounced as the rough body became more prominent in size. Similarly, the region of large triangular chip atoms in the rough body below the front side of the indenter movement gradually disappeared under this influence, as shown in Figure 12a–c. In the plowing of the small-sized indenter, some clustered chips were found in the plow path, splashing away from the surface of the rough body. Figure 13e,f shows one observed occasion, where the material exceeded the maximum tensile strength, producing a splatter at the top of the indenter. This was due to the small size of the indenter relative to the rough peak, which created a penetration-like effect on the rough body during the plowing process. The atoms of the rough peak at the top of the indenter were fractured in the continuous outward expansion of the pile-up on both sides, some of the rough peak atoms escaped outwards and the kinematic contact combined into a more significant outward movement of the chip.

Figure 14 compares the variation of the tangential and normal forces for different indenter sizes. The force curves between indenter particles with different sizes had similar mechanical characteristics. However, for a larger indenter radius, the onset of plasticity occurred at higher forces. The smaller the indenter particle radius, the earlier it reached the initial yield point of the rough peak, and the faster the tangential force entered the stable fluctuation state. Accordingly, the larger the indenter radius, the smaller the moving distance to complete the plow of the rough body. Therefore, the platform length of the stable fluctuation of the tangential force decreased with the increase in the indenter radius, as shown in Figure 14a. Compared to the indenter with different particle sizes, the indenter with different particle sizes had an almost linear decline stage in the decline stage of tangential force. This stage existed between two straight lines of the same color in the figure, related to the stable development of cracks. With the decrease in indenter radius, a longer displacement distance was required to make the rough body produce cracks. Meanwhile, the smaller indenter bore higher stress and the crack propagation was completed first. Therefore, the length of the linear decline stage was also reduced. At the same time, the more minor indenter caused a more significant shear strain rate on the localized distortion in the lattice near the contact area. Therefore, with the decrease in the indenter radius, higher stresses accelerated crack propagation. The tangential force curve with the indenter radius of 4.5 nm had the fastest decline speed in the force decline stage, confirming this view. The maximum tangential force decreased much less rapidly than the normal force as the radius of the indenter decreased, indicating an increase in the dynamic friction coefficient. After this linear descent stage, the force curve had a gradual and continuous descent stage. A similar trend of the normal force curve also showed that the plowing process was relatively consistent under different particle sizes, as shown in Figure 14b.

**Figure 15:**
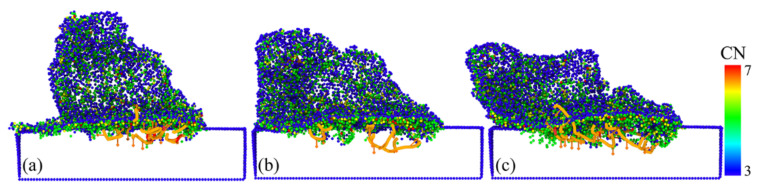
Cross-section of defect distribution after plowing by indenter with different radii of (**a**) 4.5 nm, (**b**) 6 nm and (**c**) 7 nm.

It can be seen from the comparison in Figure 15 that the depth of the subsurface damage layer in the substrate increased with the increase in the indenter radius. At the same time, the smaller the indenter size, the more asperity material was removed from the surface after plowing, as shown by the blue line comparison in Figure 15. The thickness of residual asperity on the substrate surface increased with the increase in the indenter radius, but the pile-up height of side asperity decreased.

### 3.4. The Effect of the Plowing Depth

**Figure 16:**
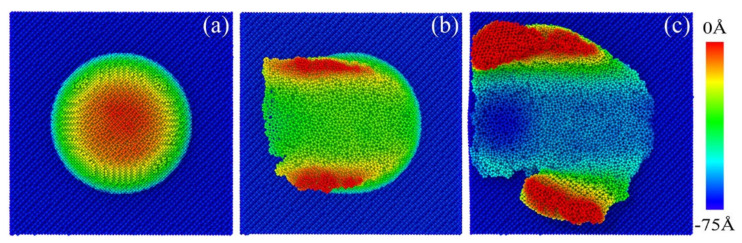
Surface morphology from different plowing depths: (**a**) no ductile ploughing was observed at 2 nm plow depth, (**b**) symmetrical plastic flow pile-up occurred at 4 nm plow depth and (**c**) significant crack propagation at 7 nm plow depth. The images were colored according to the height of the atomic z-direction.

In order to further study the effect of the plow depth on the deformation of rough GaAs, different indenter plowing depth levels were implemented, as shown in Figure 16a–c. The height of the single asperity was 7 nm and the radius of the indenter was 6 nm. A comparison of the topography of the rough peaks after plowing at different plowing depths in Figure 16 showed that when the plowing depth was small (d = 2 nm), the atomic lattice structure of the asperity with a small amount of deformation was restored and the atomic positions were maintained. Even the rough peak’s atomic position configuration hardly changed and no permanent groove could be observed after plowing, as shown in Figure 16a. However, the MD simulation still showed that GaAs deformed plastically and a good description was provided in the results. The atomic lattice in the contact region was deformed to increase its potential energy, which was stored in the rough peak lattice in the form of lattice strain energy. The lattice structure of the rough peak atom was restored, since the contact point was at the top of the rough peak. It could be found that there were many empty bonds of the atom at the top of the peak during the atomic rearrangement at the shallow plow depth, so the atom was easy to break away from the original position and re-bond with other atoms. Therefore, as shown in Figure 16a, most original positions could be maintained when the stored strain energy was released, in addition to the tendency of elastic recovery of the deformed lattice. Excess energy was released from the atomic structure at the top of the peak, which was subject to phase transformation and dislocation due to indenter action, and its atomic structure remained in the plastic state.

With the increased deformation imposed by the indenter, ductile ploughing was observed at a 4 nm plow depth and no visible cracking was detected on the surface of the asperity. The transition between elastic and plastic domains also depended on the rheology of the scratched material. In this case, the deformation of the rough body was determined by the plastic deformation, and the edges on both sides of the plow at the top of the rough peak exhibited a uniform smeared appearance, showing a continuous plastic flow. The rough peak gradually changed from hemispherical to flattened in the plowing process between the indenter and GaAs workpiece. Ductile ploughing behavior occurred at relatively small deformation levels. Note that the GaAs rough body with a plow depth of 4 nm did not undergo cracking during the plowing process, mainly elastoplastic deformation.

With the increase in the plow depth, a more significant plastic deformation occurred at the rough peak of the substrate. When the plow depth was 4 nm (Figure 16b), the chips had initially formed, indicating that the critical plow depth of chips formation was between 2 nm and 4 nm in the process of plowing. As Figure 16c showed, as the plow depth increased, a more significant elastic–plastic deformation happened in GaAs, producing more chips. At the same time, large wear debris would form. It can be seen from Figure 16b,c that as the plow depth increased, more GaAs material was removed, and the distribution of the GaAs rough peak after plowing gradually transformed from quadratic symmetry to asymmetry. This was because the plow depth became larger and the maximum stress point moved downwards from the top of the rough peak. What was essential was that the lower part of the rough peak structure was more stable than the top. At the same time, with the increase in contact area, the distribution of the stress zone was more complex. Therefore, more stress concentration points led to the opportunity of microcracks at multiple points, resulting in an asymmetric accumulation, as illustrated in Figure 16c. The structural conversion rate of the wear debris part was 94.9% at 4 nm depth, while the structural conversion rate of the wear debris part was only 61.9% at 7 nm depth. This was due to the flank-stacked atoms formed by the former formed by an extensive plastic flow. There was a partial plastic flow at the bottom and top atoms of the rough peak for the irregularly stacked atoms formed by deeper ploughing. Most of the stacked atoms on the wing sections were formed by fracture extrusion and there were many perfect cubic wurtzite structures. This was a considerable difference in deformation caused by the change of plow depth on the rough peak.

**Figure 17:**
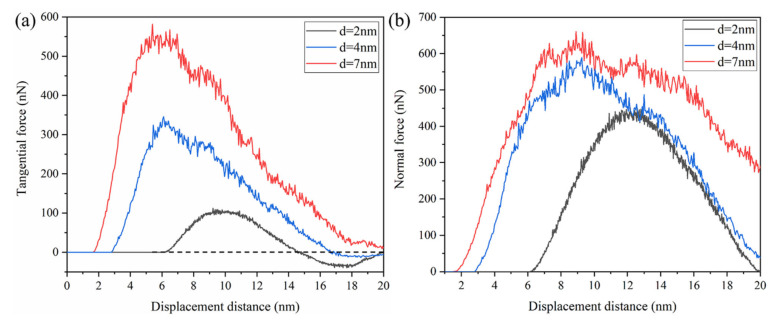
Evolution of (**a**) tangential force and (**b**) normal force at different depths of 2 nm, 4 nm and 7 nm.

The simulated force curves for GaAs under the various plow depths of 2 nm, 4 nm and 7 nm were plotted in Figure 17. The regime higher plow depth corresponded to higher friction. When the indenter was almost entirely detached from the rough peaks, the frictional force was opposing. This finding was particularly evident at a low plow depth (d = 2 nm), which was caused by the elastic recovery of the rough peaks causing the direction of the force of the indenter to coincide with the direction of motion, as shown in the black curve in Figure 17a. Moreover, the lower plow depth led to a minor plastic deformation, so more elastic strain energy was retained. Compared to the minimum plow depth (d = 2 nm), the sharp force fluctuation was caused by producing many chips at the maximum plow depth. At the same time, the normal force curve was basically symmetrical at the plow depth of 2 nm, which showed that the elasticity recovered well and the atomic position was well maintained at a small plow depth. As the plow depth decreased, the tangential force decreased much more than the normal force; thus, indicating a decrease in the dynamic friction coefficient. In the case of the 4 nm plow depth, the tangential force curve had similar mechanical characteristics to the 7 nm depth. However, when the tangential force reached the maximum value, it did not fluctuate near the stable platform, but immediately decreased due to the nucleation of dislocation loops at a 6 nm displacement distance. No platform before a sharp drop in force was observed in the tangential force curve. This was due to the smaller plow depth and the faster arrival of atoms in the contact area with an indenter to the yield limit; V. K. Jain [40] reported on this in the relevant literature.

**Figure 18:**
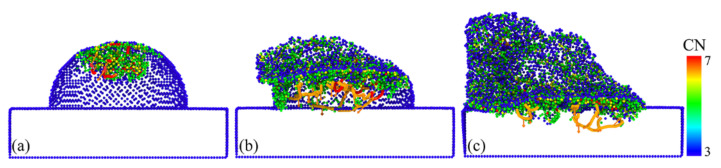
Cross-sectional image of dislocation movement and phase transformation distribution at different plowing depths of (**a**) 2 nm, (**b**) 4 nm and (**c**) 7 nm.

No chips or residual imprint were observed at a small plow depth of d = 2 nm during the low-load plow, as shown in Figure 16a. In order to determine the degree of plastic deformation, we analyzed the internal defects. The results showed that GaAs deformed plastically. The defect distribution remaining in the rough peak after plowing provided a good description, as shown in Figure 18a. Dislocation nucleations occurred inside the rough peak near the top of the surface and generated loops on the (111) slip planes. Upon plowing the rough peak, the indenter was separated from the rough peak and the deformed zone underwent incomplete elastic recovery, indicating that the plastic deformation rearrangement was irreversible and had resulted in a permanent plastic deformation. Finally, several dislocations remained on the GaAs surface upon the complete removal of the load, as illustrated in Figure 18a.

## 4. Conclusions

We used MD simulations to investigate the deformation behavior of plowing-induced single asperity GaAs. We reported that plastic deformation and surface tensile cracking jointly mediated the deformation mechanism in the asperity. Furthermore, the influence of the asperity size, indenter radius and plow depth on the deformation of the asperity was also discussed. The main conclusions were as follows:

(1) The deformation of single asperity GaAs during plowing was dominated by a combination of dislocation activity and crack propagation. Single asperity GaAs still sprouted cracks in addition to ductile behavior in deformation at the nanoscale, as well as crack propagation and further fracture. The sudden drop in the force event was found to be associated with crack generation in addition to reflecting traditional microstructural events. In all cases where cracks were generated, they formed at the intersection of (111) slip bands and were terminated at the substrate surface without extending into the substrate interior. The presence of cracks added to the variability of deformation patterns in the asperity and the ability of the asperity to resist the degree of subsurface damage to the substrate.
(2) The size of the indenter influenced the location, number and propagation of cracks and affected the type of defect and lattice integrity in the substrate. As the indenter size increased, the shielding effect of the primary emergent crack on the secondary emergent crack took effect, while being less likely to form chips in front of the indenter. The residual thickness and depth of the subsurface damage layer also increased, while the pile-up height decreased. For smaller indenter sizes, the formation and propagation of the second emergent crack would be more favorable, while the crack extension rate was higher. As the plow depth increased, the atomic pile-up of asperity gradually changed from a secondary symmetric distribution to an asymmetric distribution.

## Figures and Tables

**Table 1:** Detailed parameter settings in the MD simulation model.

Parameter	Value
Workpiece material	Gallium arsenide
Crystal structure of workpiece	Zinc-blende structure
Lattice constant	5.6532 Å
Indenter size	Diameter: 9 nm, 12 nm and 14 nm
Semispherical rough peak size	Radius: 2 nm, 4 nm and 7 nm
Plowing speed	100 m/s
Depth of plowing	2 nm, 4 nm and 7 nm
Simulation system	Microcanonical ensemble (NVE)
Algorithm	Velocity Verlet
Initial temperature	300 K
Potential for workpiece	Tersoff potential
Time step	1 fs

**Table 2:** Crystal properties of GaAs in different crystal orientations.

Orientation	Atomic Surface Density	Crystal Spacing
(001)	$\frac{2}{a^{2}}$	$\frac{a}{4}$
(110)	$\frac{2\sqrt{2}}{a^{2}}$	$\frac{\sqrt{2}a}{4}$
(111)	$\frac{8\sqrt{3}}{3a^{2}}$	$\frac{\sqrt{3}a}{4}$

## Data Availability

Section 2 described, in detail, the methods and parameters. Standard modeling and simulations are available in the open-source atomic/molecular massively parallel simulator code (LAMMPS).
